# Clinical characteristics, risk factor analysis and peripheral blood cell changes for early warning of multidrug‐resistant bacteria (MDR) infection in elderly patients

**DOI:** 10.1002/iid3.1347

**Published:** 2024-07-18

**Authors:** Yalan Nie, Yulan Zeng

**Affiliations:** ^1^ Department of Respiratory Medicine, Tongji Medical College, Liyuan Hospital Huazhong University of Science and Technology Wuhan Hubei China

**Keywords:** infection, multidrug‐resistant bacteria, prevention, risk factors

## Abstract

**Objective:**

To explore peripheral blood indicators that may serve as early indicators for multidrug‐resistant bacteria (MDR) infections in this demographic, with the goal of providing reference suggestions for the clinical prevention of MDR infections in elderly inpatients.

**Methods:**

Clinical data of patients were divided into the MDR‐infected group (*n* = 488) and the MDR‐uninfected group (*n* = 233) according to the results of drug sensitivity experiments, risk factors for MDR infection, and peripheral blood indicators related to MDR infections were analyzed using univariate and multivariate logistic regression in conjunction with the construction of a Chi‐squared automatic interaction detector (CHAID) decision tree model, considering statistical significance at *p* < .05.

**Results:**

Of 721 patients, 488 multidrug‐resistant strains were identified. Among them, with *Staphylococcus* spp. the most prevalent in 148 strains. The most frequent detection of MDR occurred in puncture fluid samples (167 cases). Univariate and multivariate regression analyses revealed that prolonged hospitalization, use of antibiotics preadmission, duration of antibiotics, invasive procedures or recent surgery, and coexisting lung disease were independent risk factors for contracting MDR. Subsequent analysis comparing the aforementioned influences with peripheral blood cells revealed associations between the number of antibiotic treatment days and increased neutrophil‐to‐lymphocyte ratio (NLR), platelet count‐to‐lymphocyte ratio (PLR), neutrophils, decreased lymphocytes, and increased eosinophils; preadmission antibiotic use correlated with increased PLR, NLR, neutrophils, and decreased lymphocytes; and invasive manipulation or surgery correlated with increased PLR and NLR.

**Conclusions:**

Elevated NLR, PLR, neutrophils, lowered lymphocytes, and eosinophils may serve as early indicators of MDR infections in elderly hospitalized patients.

## Graphical abstract

The manuscript presents fresh findings indicating that peripheral blood markers may act as early indicators of multidrug‐resistant (MDR) infections in elderly hospitalized patients. Alterations in neutrophil‐to‐lymphocyte ratio (NLR) and platelet count‐to‐lymphocyte ratio (PLR), as well as neutrophils, lymphocytes, and eosinophils, may also act as early indications of MDR infections in this population.

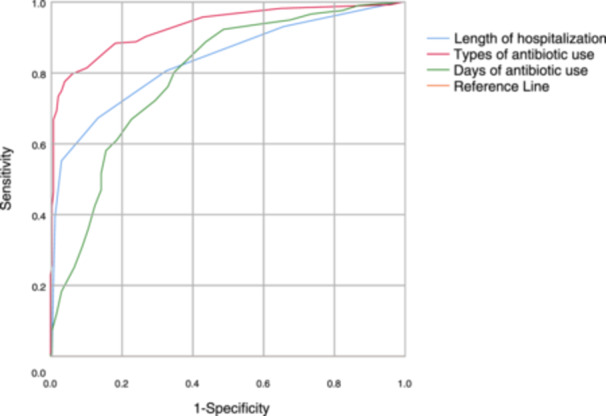

## 1 INTRODUCTION

The advent of antibiotics represents a landmark in medical history, revolutionizing healthcare practices and profoundly enhancing human well‐being. Despite these advancements, microorganisms are continually evolving, giving rise to the rapid emergence of drug‐resistant strains, thereby posing significant challenges to the global healthcare system.^1^, ^2^

In 1950, resistance to sulfonamides was observed in 80% to 90% of Shigella isolates in Japan. Subsequently, resistance to chloramphenicol, streptomycin, and tetracycline was identified in patients. Some strains of Shigella developed resistance to all four of these antimicrobials, as has been reported in various studies.^3^, ^4^

The escalating presence of multidrug‐resistant (MDR) microorganisms has garnered increased public attention. Isolates resistant to at least three classes of potentially effective antimicrobial agents were categorized as MDR bacteria. Within these isolates, those resistant to all except one or two classes of effective agents (excluding Fosfomycin) were further classified as extensively drug‐resistant.^5^

Owing to the annually increasing resistance among various microbial infectious agents to multiple antimicrobial drugs, the constant evolution of drug resistance mechanisms, and the consequent decline in the cure rates of infectious diseases, previously recognized therapeutic standards for specific pathogenic bacteria have become obsolete. Drug‐resistant microbial infections have emerged as a complex issue in global public health.^6^

For instance, methicillin‐resistant *Staphylococcus aureus* (MRSA) has been extensively studied and found to be refractory. Common clinical MDR also encompasses newly emerged strains, such as ultra‐broad‐spectrum β‐lactamase‐resistant *Klebsiella pneumoniae* and *Escherichia coli*; carbapenem‐resistant *Enterobacteriaceae* and *Acinetobacter baumannii*.^7^, ^8^

The proliferation of these drug‐resistant bacteria leads to suboptimal disease treatment, elevated morbidity and mortality rates, extended treatment duration, and escalated treatment costs. Moreover, it amplifies the psychological distress experienced by both patients and their families, as well as healthcare professionals.^1^

The evolution of MDR microorganisms is a natural occurrence; however, deficient infection prevention, inadequate control measures, and improper use of antimicrobial drugs can foster the emergence and rapid dissemination of such resistant strains. Early identification of targeted populations and preemptive planning of tailored treatment regimens can effectively diminish the incidence of MDR bacterial infections in hospitalized patients.^9^, ^10^

Elderly patients, with their diminished physical function and compromised immune defense, are particularly susceptible to MDR infections. Such infections not only exacerbate existing underlying conditions but also contribute to complications, with potential progression to deterioration or death. Therefore, immediate and efficacious indicators are essential for the prevention and management of MDR infections in this demographic. Despite this necessity, existing studies, mostly retrospective, present delayed results and lack investigations into the early warning indicators for MDR infections in elderly patients, thus failing to provide early risk identification.^11^, ^12^, ^13^, ^14^, ^15^ This study integrates the characteristics of previous retrospective analyses to explore the risk factors for MDR infection, augmenting this analysis with the Chi‐squared automatic interaction detector (CHAID) decision tree prediction model. This model automatically isolates major risk factors, facilitating the comparison of changes in peripheral blood cells in patients infected with MDR against these primary risk factors, to identify potential early warning indicators. This approach aims to furnish a scientifically robust reference for the prevention and treatment of MDR infections in elderly hospitalized patients.

## 2 METHODS

### 2.1 Subjects

The study involved elderly patients admitted to Liyuan Hospital, Tongji Medical College, Huazhong University of Science and Technology, China, from January 2021 to June 2023. Participants were divided into two groups: those infected with multidrug‐resistant organisms (*n* = 488), and those not infected with multidrug‐resistant organisms (*n* = 233). The categorization was based on the results of drug sensitivity experiments conducted by the hospital's Laboratory Department. This investigation conformed to the ethical principles outlined in the Declaration of Helsinki and received approval from the Liyuan Hospital, Tongji Medical College, Huazhong University of Science and Technology Medical Institutional Review Board ethics committee. IRB ID:[2023]IEC (RYJ008).

### 2.2 Inclusion criteria

Participants were required to meet the MDR diagnostic criteria for multidrug‐resistant bacteria: Isolates resistant to at least three classes of potentially effective antimicrobial agents^5^, ^16^; have complete laboratory and clinical data; and not be infected with multidrug‐resistant bacteria before admission.

### 2.3 Exclusion criteria

Individuals were excluded if they had missing information in medical records; were diagnosed with malignant tumors or hematological diseases; were in ICU units; refused to cooperate with examination and follow‐up; or had been infected with multidrug‐resistant bacteria before admission.

### 2.4 Study content

(a) General information of patients: Information collected included age, gender, departmental distribution, and species of drug‐resistant microorganisms. (b) Laboratory indicators: These encompassed routine blood count, neutrophil‐to‐lymphocyte ratio (NLR), and platelet count‐to‐lymphocyte ratio (PLR), calculated by formula; and biochemical indicators such as ultrasensitive C‐reactive protein, fibrinogen, fasting glucose, albumin level, globulin level, and albumin‐to‐globulin ratio, also calculated by formula. (c) Medical records: Data retrieved included length of hospitalization, comorbidities (e.g., lung infection, cardiovascular disease, diabetes mellitus), antibiotic use (including preadmission use, type, and duration of treatment), and information regarding invasive operations and recent surgeries.

### 2.5 Statistical analysis

All experimental data were subjected with SPSS 26.0 (SPSS Inc.,). Measurement information not conforming to normal distribution was represented as M (P25, P75), and group comparisons were made using the Mann–Whitney *U* test. Count information was denoted as the number of cases or percentage, with group comparisons conducted via Pearson's *χ* ^2^ test.

Measured data profiles linked to MDR infection, and then grouped these data profiles according to the cut‐off values that were established using receiver operating characteristic (ROC) curves. On the grouped variables, univariate logistic regression analysis was carried out, and multivariate logistic regression analysis was applied to those that demonstrated *p* < .05 in the univariate logistic regression study. Next, the CHAID decision tree model was constructed utilizing the multivariate logistic regression analysis variables indicated above with *p* < .05.

To identify the key risk factors associated with MDR, variables that had a *P*‐value of less than 0.05 in the multivariate logistic regression analyses above were incorporated into this model. These key risk factors were then compared to the control group and the elevated/reduced group in peripheral blood cells, respectively, using binary logistic regression and Pearson's *χ* ^2^ test. Differences were deemed statistically significant at *p* < .05.

## 3 RESULTS

### 3.1 General data

Table 1 shows the fundamental characteristics of 488 elderly individuals infected with MDR. The patients' median age was 74 years, with a range of 60–100 years, with 61.7% being male and 38.3% being female. According to the statistics on pre‐existing underlying conditions, the most common categories were cardiovascular diseases in 290 patients (59.4%), lung infection in 262 patients (53.7%), and diabetes mellitus in 318 patients (71.3%). The number of patients who had taken antibiotics previous to admission was 390 (79.9%), and the number of patients who had undergone invasive operations/recent surgery was 343 (70.3%) (Table 1). Statistical analyses revealed no significant differences in age and gender between the two groups (Table 2).

**Table 1:** Baseline data.

Baseline data	Infected MDR (*n* = 488)
Male (*n*, %)	301,61.7%
Age (range)	74 (60–100)
Use of antibiotics before admission (*n*, %)	390,79.9%
Invasive operations/recent surgery(*n*, %)	343,70.3%
Cardiovascular disease (*n*, %)	290,59.4%
Lung infection (*n*, %)	262,53.7%
Diabetes mellitus (*n*, %)	318,71.3%
Platelet,10^9/L (IQR)	220.50 (163.00, 298.00)
Neutrophils,10^9/L (IQR)	5.27 (3.57, 8.01)
Lymphocytes,10^9/L (IQR)	1.27 (0.90, 1.68)
Eosinophils,10^9/L (IQR)	0.11 (0.07, 0.20)
NLR (IQR)	4.15 (2.51, 7.43)
PLR (IQR)	183.89 (124.13, 263.64)
Fasting glucose, mmol/L (IQR)	6.81 (5.34, 9.32)
Albumin to globulin ratio (IQR)	0.95 (0.78, 1.18)
Fibrinogen, g/L (IQR)	3.41 (2.66, 4.14)
C‐reactive protein, mg/L (IQR)	11.50 (3.58, 37.30)
Length of hospitalization (IQR)	19.00 (13.00, 25.00)
Type of antibiotics used (IQR)	4.00 (2.00, 6.00)
Days of antibiotics used (IQR)	12.00 (3.00, 20.00)

Abbreviations: IOR, quartile deviation; MDR, multidrug‐resistant; NLR, neutrophil‐to‐lymphocyte ratio; PLR, platelet count‐to‐lymphocyte ratio.

**Table 2:** General data.

Group	Infected MDR	Uninfected MDR	*χ* ^2^/Z	*P*‐value
Sex (Male/Famale)	301/187	129/94	.941	.332
Age (Range)	74 (60–100)	75 (60–102)	.103	.918

Abbreviation: MDR, multidrug‐resistant.

Types of pathogenic microorganisms detected and sources of samples: Of 721 patients, 488 multidrug‐resistant strains were identified. Among them, Gram‐positive strains were found in 208 cases, with *Staphylococcus* spp. the most prevalent in 148 strains. Gram‐negative strains were detected in 280 cases, with *Fusobacterium* spp. the most common, accounting for 79 strains. The most frequent detection of MDR occurred in puncture fluid samples (167 cases), followed by sputum samples (124 cases) (Table 3).

**Table 3:** Types and sources of multidrug‐resistant microorganisms.

Microorganisms	MDR	Focal secretions	Puncture fluid	Urine	Sputum	Blood	Tissue
*Escherichia*	57	9	18	18	2	5	5
*Aspergillus*	19	6	8	2	–	–	3
*Fusobacterium*	79	10	29	4	31	1	4
*Enterobacteriaceae*	14	5	5	–	2	1	1
*Morganella* spp.	11	1	10	–	–	–	–
*Serratia marcescens*	10	2	5	–	2		1
*Citrobacter*	9	3	2	1	–	1	2
*Pseudomonas* spp.	50	1	7	2	39	–	1
*Klebsiella*	31	2	4	8	15	2	–
*Streptococcus*	19	–	3	–	3	13	–
*Enterococci*	41	8	13	12	–	3	5
*Staphylococcus*	148	35	63	1	30	9	10

Abbreviation: MDR, multidrug‐resistant.

### 3.2 Clinical data analysis

Peripheral blood changes in patients infected with MDR and those not infected with MDR. A comprehensive examination was undertaken to compare the peripheral blood cell characteristics between groups infected with multidrug‐resistant infections and those uninfected. The analysis revealed that the *p*‐value for both monocyte and platelet counts was greater than 0.05, deeming them not statistically significant. This result underlines the absence of a substantial relationship between monocytes, platelets, and illnesses attributable to MDR strains.

**Figure 1:**
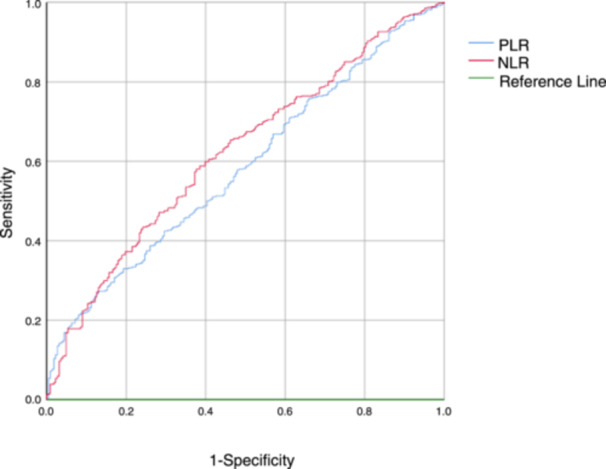
ROC curves of PLR and NLR with multidrug‐resistant bacterial infections. NLP, neutrophil‐to‐lymphocyte ratio; PLR, platelet count‐to‐lymphocyte ratio; ROC, receiver operating characteristic.

Contrastingly, the *p*‐value for neutrophils, lymphocytes, eosinophils, PLR, and NLR was less than 0.05. The statistical significance in this difference delineates the association of these parameters with MDR infection. For further categorization, patients were aligned according to the optimal cut‐off value from the ROC curve (as depicted in Figure 1). Those exceeding the cut‐off value were allocated to the higher group (e.g., PLR > 274.537 was classified as the PLR higher group; NLR > 3.979 as the NLR higher group).

Neutrophils, lymphocytes, and eosinophils were further segregated into high (above normal range), low (below normal range), and control groups (within normal range) based on the normal range defined by the hospital laboratory. A one‐way comparison among the infected MDR group, the uninfected MDR group, and the control group in the higher or lower categorization revealed statistically significant differences (*p* < .05) among the infected MDR groups (Table 4).

**Table 4:** Peripheral blood changes in patients infected with MDR and those not infected with MDR.

Group	*χ* ^2^ test		Binary logistics regression	
	*χ* ^2^	*P*	OR (95% CI)	*P*
High PLR	17.665	<0.001	2.042 (1.393‐2.994)	<.001
High NLR	24.462	<0.001	2.228 (1.610‐3.083)	<.001
High neutrophils	12.239	<0.001	1.829 (1.301‐2.570)	.001
Low lymphocytes	7.917	0.005	1.621 (1.156‐2.271)	.005
High eosinophils	10.638	0.001	0.115 (0.024‐0.545)	.006

Abbreviations: Cl, confidence interval; MDR, multidrug‐resistant; NLR, neutrophil‐to‐lymphocyte ratio; OR, odds ratio; PLR, platelet count‐to‐lymphocyte ratio.

The Manny Whitney test and binary logistic regression both indicated a *p* > .05 for the albumin‐to‐globulin ratio, suggesting no significant correlation with MDR infections. Conversely, fasting glucose, fibrinogen, C‐reactive protein, types and duration of antibiotics used, revealed a statistically significant *p* < .05 when analyzed by both statistical methods (Table 5).

**Table 5:** Risk factors for MDR infection.

Contents	Mann–Whitney *U* test			Binary logistics regression	
	M (P25, P75)	Z	*P*‐value	OR (95%CI)	*P*‐value
Fasting glucose (mmol/L)	6.81 (5.34, 9.32)	−3.542	<0.001	0.949 (0.915, 0.986)	.006
Albumin to globulin ratio	0.95 (0.78, 1.18)	0.676	0.499	1.255 (0.741, 2.125)	.399
Fibrinogen (g/L)	3.41 (2.66, 4.14)	−7.148	<0.001	0.498 (0.420, 0.591)	<.001
C‐reactive protein (mg/L)	11.50 (3.58, 37.30)	−2.763	0.006	0.993 (0.989, 0.997)	<.001
Type of antibiotics used	4.00 (2.00, 6.00)	14.419	<0.001	2.211 (1.944, 2.514)	<.001
Days of antibiotics used	12.00 (3.00, 20.00)	18.386	<0.001	1.424 (1.345, 1.506)	<.001
Length of hospitalization	19.00 (13.00, 25.00)	12.448	<0.001	1.182 (1.148, 1.217)	<.001

Abbreviations: MDR, multidrug‐resistant; OR, odds ratio.

**Figure 2:**
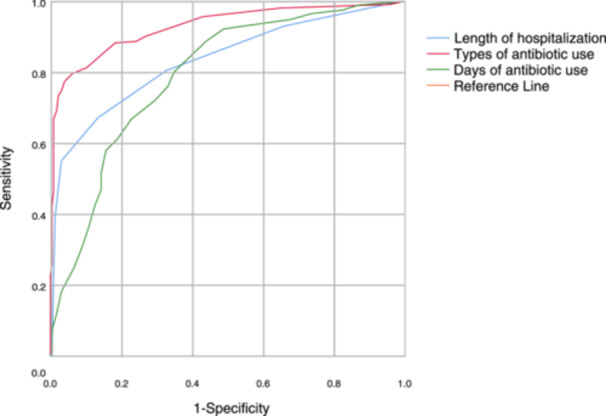
ROC curves for type of antibiotic use, days of antibiotic use, and length of hospitalization. ROC, receiver operating characteristic.

ROC curve analysis of the working characteristics of the subjects revealed optimal cutoff values for types of antibiotics used, number of days of antibiotics used, and length of hospitalization as 3.50, 8.50, and 18.50, respectively. Patients were subsequently categorized into two groups based on these ROC curve values: Types of antibiotic use >3.5 signified an increased variety of antibiotic therapy; Days of antibiotic therapy >8.5 indicated extended antibiotic treatment; Length of hospitalization >18.5 was considered as prolonged hospitalization (Figure 2).

Chi‐square test results demonstrated that the numbers of patients in the infected MDR group with increased types of antibiotic therapy, extended days of antibiotic treatment, prolonged length of hospitalization, preadmission antibiotic use, invasive operations or recent surgery, and comorbidities such as pulmonary disease, cardiovascular and cerebrovascular disease, or diabetes mellitus were significantly higher than those in the uninfected MDR group. A *p*‐value of less than 0.05 indicated a significant association with MDR bacterial infection (Table 6).

**Table 6:** The results of the *χ* ^2^ test.

Group	Infected MDR	Uninfected MDR	*χ* ^2^	*P*‐value
Increased types of antibiotic therapy	328	193	29.218	<.001
Increased days of antibiotic treatment	389	209	22.471	<.001
Prolonged length of hospitalization	326	162	118.6	<.001
Use of antibiotics before admission	390	18	323.07	<.001
Invasive operations/recent surgery	343	59	119.66	<.001
Pulmonary disease (yes)	262	34	93.082	<.001
Cardiovascular (yes)	290	160	10.003	.002
Diabetes mellitus (yes)	318	91	37.162	<.001

Abbreviation: MDR, multidrug‐resistant.

Further, multifactorial logistic regression analysis incorporated these factors and identified prolonged hospitalization, use of antibiotics before admission, the number of days on antibiotics, invasive operations or recent surgery, and comorbidities with lung disease as independent risk factors for MDR infection in elderly patients (Table 7).

**Table 7:** Univariate and multivariate logistic regression of risk factors for MDR infection.

Group	Univariate logistic regression		Multivariate logistic regression	
	OR (95%CI)	*P*‐value	OR (95%CI)	*P*‐value
Increased types of antibiotic therapy	13.188 (8.591, 20.245)	<0.001	0.765 (0.284, 2.061)	.596
Increased days of antibiotic treatment,	58.659 (32.701, 105.222)	<0.001	61.049 (20.601, 180.916)	<.001
Prolonged length of hospitalization	0.147 (0.102, 0.212)	<0.001	6.353 (2.883, 13.996)	<.001
Use of antibiotics before admission (yes)	45.323 (26.670, 77.023)	<0.001	45.068 (18.46, 110.028)	<.001
Invasive operations/recent surgery (yes)	6.575 (4.609, 9.380)	<0.001	10.667 (4.257, 26.731)	<.001
Pulmonary disease (yes)	6.444 (4.293, 9.673)	<0.001	3.1 (1.404, 6.845)	.005
Cardiovascular (yes)	3.720 (2.640, 5.242)	<0.001	1.138 (0.469, 2.759)	.775
Diabetes mellitus (yes)	1.290 (0.931, 1.786)	0.126	‐	‐

Abbreviations: CI, confidence interval; OR, odds ratio.

### 3.3 Predictive modeling

**Figure 3:**
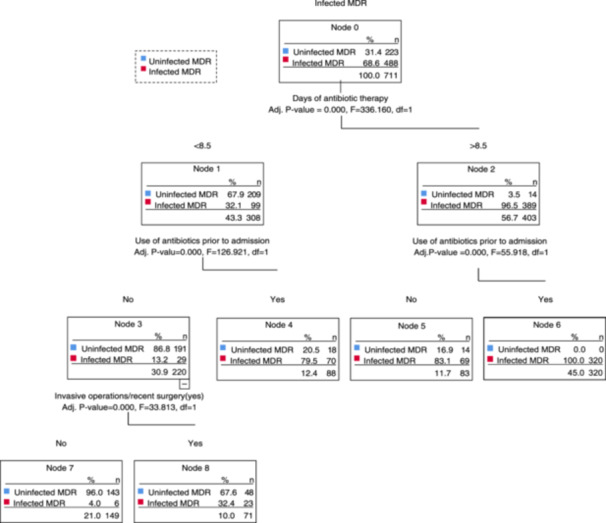
Risk factor prediction model diagram for MDR infection. MDR, multidrug‐resistant.

In our study, we incorporated factors with a *p*‐value of less than 0.05 from the one‐way logistic regression analysis into the CHAID decision tree model. The findings demonstrated that the number of days of antibiotic use emerged as the primary predictor, with strong associations with MDR infection. Notably, an interaction was observed between the duration of antibiotic use and prior admission antibiotic treatment. For durations less than 8.5 days, the probability of MDR infection in patients previously treated with antibiotics was 79.5%, contrasted with a 13.2% likelihood in elderly patients without prior antibiotic use. In cases exceeding 8.5 days of antibiotic treatment, the probability of MDR infection was markedly lower among those who had not received antibiotics before admission. Furthermore, an interaction was detected between prior antibiotic use and invasive procedures or surgeries performed during hospitalization; the probability of contracting MDR was 32.4% for patients without preadmission antibiotic treatment, which further reduced to 4% if they were not subjected to invasive procedures or surgeries after admission (see Figure 3).

**Figure 4:**
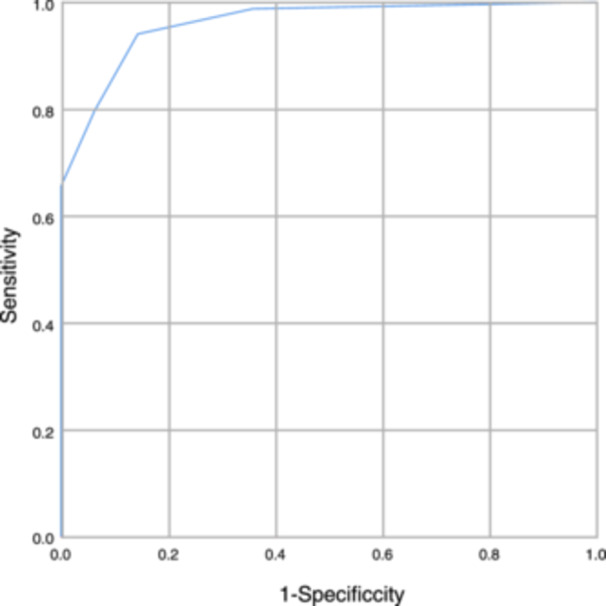
ROC curve of decision tree prediction probability. ROC, receiver operating characteristic.

The decision tree model's predictive capability was supported by an area under the ROC curve of 0.961 (*p* < .001), signifying that this model could accurately forecast the risk of MDR infection in elderly hospitalized patients. This conclusion was drawn based on the plotted ROC curves reflecting the subjects' work characteristics and the validation results of the decision tree model (see Figure 4).

Analysis of independent risk factors and peripheral blood cell alterations in elderly hospitalized patients with MDR infection: Based on the preceding observations, we identified and investigated three pivotal independent risk factors for contracting MDR infection: the duration of antibiotic therapy, the administration of antibiotics before admission, and the execution of invasive operations or surgeries. A meticulous comparison of these risk factors with the higher/lower groupings in peripheral blood cells versus the control group was performed.

The resultant analysis elucidated specific associations. Namely, the number of days of antibiotic treatment corresponded with a higher PLR (*p* = .017), NLR (*p* = .018), neutrophils (*p* = .024), eosinophils (*p* = .008), and lower lymphocytes (*p* = .012). The administration of antibiotics before hospital admission was associated with higher PLR (*p* = .004), NLR (*p* < .001), neutrophils (*p* = .006), and lower lymphocytes (*p* = .008). Furthermore, the engagement in invasive manipulation or surgery correlated with higher PLR (*p* = .016) and NLR (*p* = .014), as delineated in Table 8.

**Table 8:** Analysis of independent risk factors and peripheral blood cell alterations in patients with MDR infection.

Group	High PLR		High NLR		High neutrophils		Low lymphocytes		High eosinophils	
	*χ* ^2^	*P*‐value	*χ* ^2^	*P*‐value	*χ* ^2^	*P*‐value	*χ* ^2^	*P*‐value	*χ* ^2^	*P*‐value
Increased types of antibiotic therapy	5.653	0.017	5.638	0.018	5.06	0.024	6.26	0.012	7.068	0.008
Use of antibiotics before admission	8.408	0.004	16.93	<0.001	7.588	0.006	7.13	0.008	3.829	0.051
Invasive operations/recent surgery	5.846	0.016	6.008	0.014	3.552	0.061	0.06	0.808	2.202	0.138

Abbreviations: MDR, multidrug‐resistant; NLR, neutrophil‐to‐lymphocyte ratio; PLR, platelet count‐to‐lymphocyte ratio.

## 4 DISCUSSION

### 4.1 Analysis of general information

Our investigation encompassed clinical data from 721 patients collected between January 2021 and June 2023, across 11 distinct departments within the hospital. These patients were stratified into two groups: those infected with MDR bacteria and those uninfected. We omitted patients with systemic diseases, malignant tumors, hematological disorders, and those hospitalized in intensive care units (ICUs), whose clinical indices could be substantially influenced by their primary or secondary ailments. The exclusion criteria further encompassed patients previously infected with MDR before admission, as their clinical data during uninfected periods could not be procured and, therefore, were unsuitable for statistical analysis. Upon omitting these confounding elements, our analysis discerned that neither gender nor age exhibited a substantial correlation with MDR infection. We identified 12 MDR genera in this study, with *Staphylococcus* spp. being the most widespread, succeeded by *Fusobacterium* spp. Notably, we ascertained that the rate of MDR infection among surgical inpatients was markedly higher compared to that in medical departments. *Staphylococcus* spp. (*n* = 98) was predominant in surgical departments, followed by *Fusobacterium* spp. (*n* = 44), whereas medical departments were chiefly characterized by *Staphylococcus* spp. (*n* = 50), and *Pseudomonas* spp. (*n* = 40). Puncture fluid and sputum samples yielded the highest detections of MDR. These findings, underlined by regional and population distinctions, contrast with the distribution of drug‐resistant bacteria as observed in the Clinical University Hospital of Zaragoza in Spain by Barrasa‐Villar et al., where *E. coli* prevailed, followed by *S*. *aureus* and *Pseudomonas aeruginosa*.^17^

### 4.2 Peripheral blood and MDR

The routine blood test typically constitutes the initial examination upon patient admission and ranks among the more expeditious diagnostics for obtaining test results. Upon the invasion of pathogenic microorganisms, blood cells undergo immediate alterations, with neutrophils acting as the primary responsive agents against damage and infection in the body's tissues, thereby fulfilling a crucial protective role.^18^, ^19^ Lymphocytes, as an integral component of the body's immune defense system, serve as an essential barrier for the organism. Conversely, neutrophils, being the principal phagocytes of the organism and responsible for the eradication of extracellular pathogens, possess both a vital defensive position against pathogenic infections and a paradoxical capacity to host certain pathogens within their cells. This duality promotes their cytolysis, potentially leading to the organism's acquisition of infections.^18^, ^20^ During bacterial invasion of the bloodstream, NLR typically manifests through elevated neutrophils and diminished lymphocytes, culminating in an increased NLR value, a finding congruent with the results of this study. However, this contrasts with the findings of Zhou et al., concerning the interrelation between multidrug resistance and NLR in patients with hospital‐acquired pneumonia, where they indicated that a decrease in NLR prognosticates the risk of MDR *P. aeruginosa* infection.^21^ Such discrepancies may be attributed to variations in pathogen‐infected patient cohorts, hospitalization and treatment protocols, or the virulence of the pathogens. Nevertheless, the significance of NLR in the early risk screening for MDR infection remains unambiguous. Recent investigations have corroborated the capacity of NLR and PLR to serve as next‐generation inflammatory markers, reflecting the severity of systemic infections and exhibiting robust correlations with the prognoses of several neoplastic diseases.^22^, ^23^, ^24^ The Miura et al. investigation established that, compared to tumor node metastasis classification, increased neutrophil–lymphocyte ratio demonstrated a greater predictive value in the clinical characteristics of malignant tumors.^25^

### 4.3 Risk factor analysis

In our study, we initially screened risk factors associated with MDR infection and performed a comprehensive univariate and multifactorial regression analysis. Based on prior research, such as the study by Dubský et al., that affirmed higher‐than‐normal blood glucose levels as an essential risk factor for multidrug resistance. This is consistent with the results of our univariate analysis but is not an independent risk factor for MDR infection. This may be related to the selection of the target population.^26^ Our conclusions pinpointed prolonged hospitalization, preadmission antibiotic use, extended days on antibiotics, recent invasive manipulation/surgery, and comorbidities with lung disease as independent risk factors for MDR infection in elderly patients. This contradicted some reports, such as one by Jia et al., where healthcare‐associated infections caused by certain MDRs did not prolong hospital stay, and other studies where minimal or no effects of MDR on extended hospital length of stay were estimated in both ICU patients and throughout the hospital.^27^, ^28^

Our findings also resonate with the broader scientific understanding of the relationship between antibiotic use and bacterial resistance. The study by Hyle et al. underscores that the clinical focus should be placed on the threshold of therapeutic doses of antibiotics. Their conclusion, suggesting that antibiotic usage for less than 24 h might minimize the risk of resistance, aligns with our results.^29^ We discerned that prior antibiotic usage and the administration of antibiotics for more than 8.5 days independently predisposed patients to contracting MDR infections. Correspondingly, various studies substantiated that extended use of particular antibiotics curtails the recurrence of chronic obstructive pulmonary disease (COPD), mitigates its exacerbation frequency, and augments the prognosis concerning patients' quality of life. Nevertheless, the implications of antibiotic‐induced adverse events and the concomitant emergence of microbial resistance necessitate further investigation.^30^, ^31^ A robust association between surgical or invasive procedures and MDR has been observed. Several studies have corroborated that invasive procedures or recent surgeries are potently linked to bacterial infections.^32^, ^33^ Patients who contract COVID‐19 are more vulnerable to coinfections because the virus compromises their immune systems and reduces their lymphocyte counts. In keeping with our findings, Rawson et al. showed that patients with COVID‐19 are more likely to undergo invasive therapies like central venous catheters, automated ventilation, and urinary catheters. This greatly increases the risk of acquiring MDR strains of the organisms.^34^

As an illustration, post‐lung transplantation patients manifest an elevated incidence of gram‐negative MDR infection, and MDR‐related infections are recognized as significant contributors to escalated mortality subsequent to transplantation.^35^ Moreover, lung infections, prevalently encountered among elderly hospitalized patients, can amplify the severity of their primary diseases and elevate mortality rates, due to their vulnerability to MDR and suboptimal choices in empirical antibiotic therapy.^36^

### 4.4 CHAID decision tree model

To elucidate the principal determinants, we incorporated a decision tree predictive model. The findings indicate that the number of days of antibiotics at the time of treatment serves as the primary influence on MDR infection, succeeded by preadmission antibiotic usage. Despite identifying the salient influencing factors, our study's retrospective nature precluded these from being prospective indicators. Consequently, we pursued a supplementary investigation to explore more immediate and accessible indicators.

We discerned a correlation between the ratios of NLR, PLR, neutrophils, lymphocytes, and eosinophils, and three pivotal risk factors: duration of antibiotic treatment, preadmission antibiotic usage, and invasive procedures/surgeries. Furthermore, associations were identified between these hematological parameters and MDR infections, specifically elevated PLR, neutrophil count, eosinophils, NLR, and decreased lymphocytes. These associations were correspondingly linked with the number of days of antibiotic treatment, usage of antibiotics before admission, invasive procedures/surgeries, and MDR infection. The NLR demonstrates associations with a myriad of diseases. For instance, NLR serves as an independent predictor for major adverse cardiovascular events and also prognosticates hospital‐acquired bacterial infections in patients suffering from decompensated liver cirrhosis.^37^, ^38^ When evaluating the inflammatory and nutritional condition of drug‐ or multidrug‐resistant tuberculosis patients, NLR, PLR, and MLR are affordable and straightforward prognostic markers. Patients suffering from drug‐ or multidrug‐resistant tuberculosis have an increased chance of dying when their levels of NLR and PLR are elevated.^39^

Concurrently, the PLR is correlated with myocardial infarction or the prognostic status of immunotherapy in gastric cancer patients and has been identified as a predictive factor for systemic inflammatory response syndrome and sepsis.^40^, ^41^, ^42^ Intubation and elevated NLR were associated with longer hospital stays and mortality in 142 Saudi Arabian Coronavirus disease (COVID‐19) critical care unit admissions. Coinfection with MDR bacterial strains increases the likelihood of problems and an extended hospital stay for patients receiving critical care unit treatment.^43^ The NLR and PLR are used together or separately for the assessment of a wide range of diseases, such as malignancies (including hematological malignancies), respiratory diseases, gastrointestinal and cardiovascular diseases (acute coronary syndromes, cerebral hemorrhage), systemic diseases, and more recently, psychiatric disorders, such as COVID‐19, obsessive‐compulsive disorders, as well as for assessing the severity of disease in pediatric patients, and have clinical predictive value for the diagnosis of early‐onset neonatal sepsis and bacterial infectious pneumonia in children also have clinical predictive value, with higher values correlating with disease severity and prognosis.^44^, ^45^, ^46^, ^47^, ^48^ Though limited studies have acknowledged PLR as a risk predictor, research conducted by Ayeni et al. has verified that both NLR and PLR function as indicators for diagnosing appendicitis and determining its severity in children; these findings align with our conclusion that PLR and NLR may be vital markers for the early prevention of MDR infections in elderly hospitalized patients.^49^ It has also been demonstrated that NLR and PLR are newly emerging markers that are highly correlated with a bad prognosis in individuals suffering from an acute exacerbation of COPD. Higher NLR levels were found to be independently linked in their study to pneumonic acute exacerbation caused by *K. pneumonia*, a carbapenem‐resistant bacteria.^50^

In the context of elderly patients manifesting elevated NLR, PLR, neutrophil count, diminished lymphocytes, and increased eosinophils, attention must be concentrated on the duration of antibiotic therapy at the time of admission. For patients displaying elevated NLR, PLR, neutrophil count, and decreased lymphocytes, consideration must be given to whether antibiotics were administered before admission and the length of subsequent antibiotic therapy. Moreover, for patients with increased NLR and PLR, the focus should be directed towards the span of antibiotic treatment, regardless of prior antibiotic therapy, and efforts must be made to minimize the utilization of invasive procedures.

### 4.5 Advantages and limitations

The merits of our study reside in its rigorous design and the employment of a CHAID decision tree prediction model founded on logistic regression analysis to discern major risk factors for MDR infection in elderly hospitalized patients. This was further augmented by the exploration for early warning biomarkers for MDR infection, synchronized with peripheral blood cell variations. Unlike many recent investigations that solely targeted risk factor identification, our study also ventured into the domain of early warning indicators, enabling patient stratification for personalized therapeutic management at admission, thereby augmenting the strengths of our research. Nonetheless, certain limitations persist in our study. The patient data assembled were confined to our cohort, and pertaining to antibiotic therapy, details concerning the dosage and type of antibiotics administered during treatment were not captured, marking an area where the study could be enhanced.

In conclusion, surgical and specific invasive units must remain vigilant to MDR infections. Factors such as extended hospitalization, preadmission antibiotic use, the number of days on antibiotics, invasive operations or recent surgeries, and comorbidities with lung diseases emerge as independent risk factors for MDR infections in elderly patients. Furthermore, altered levels of NLR, PLR, neutrophils, lymphocytes, and eosinophils may serve as early indicators for MDR infections within this demographic.

## AUTHOR CONTRIBUTIONS

**Yalan Nie**: Conceptualization; data curation; formal analysis; investigation; methodology; resources; software; writing—original draft; writing—review & editing. **Yulan Zeng**: Conceptualization; resources; visualization; writing—review & editing.

## Data Availability

The data that support the findings of this study are available from the corresponding author upon reasonable request.
